# Delayed intracranial hemorrhage after head trauma seems rare and rarely needs intervention—even in antiplatelet or anticoagulation therapy

**DOI:** 10.1186/s12245-023-00530-z

**Published:** 2023-09-04

**Authors:** Henrik Bergenfeldt, Jakob Lundager Forberg, Riikka Lehtinen, Ebba Anefjäll, Tomas Vedin

**Affiliations:** 1 Clinical Research Centre, Department of Clinical Sciences, Skåne University Hospital, Lund University, Box 50332, 20213 Malmö, Sweden; 2 Clinical Sciences, Helsingborg General Hospital, Lund University, Svartbrödragränden 3-5, 25187 Helsingborg, Sweden

**Keywords:** MESH, Brain injuries, Traumatic intracranial hemorrhages, Traumatic anticoagulant tomography, X-ray computed

## Abstract

**Background:**

Traumatic brain injury causes morbidity, mortality, and at least 2,500,000 yearly emergency department visits in the USA. Computerized tomography of the head is the gold standard to detect traumatic intracranial hemorrhage. Some are not diagnosed at the first scan, and they are denoted “delayed intracranial hemorrhages. ” To detect these delayed hemorrhages, current guidelines for head trauma recommend observation and/or rescanning for patients on anticoagulation therapy but not for patients on antiplatelet therapy. The aim of this study was to investigate the prevalence and need for interventions of delayed intracranial hemorrhage after head trauma.

**Methods:**

The study was a retrospective review of medical records of adult patients with isolated head trauma presenting at Helsingborg General Hospital between January 1, 2020, and December 31, 2020. Univariate statistical analyses were performed.

**Results:**

In total, 1627 patients were included and four (0.25%, 95% confidence interval 0.06–0.60%) patients had delayed intracranial hemorrhage. One of these patients was diagnosed within 24 h and three within 2–30 days. The patient was diagnosed within 24 h, and one of the patients diagnosed within 2–30 days was on antiplatelet therapy. None of these four patients was prescribed anticoagulation therapy, and no intensive care, no neurosurgical operations, or deaths were recorded.

**Conclusion:**

Traumatic delayed intracranial hemorrhage is rare and consequences mild and antiplatelet and anticoagulation therapy might confer similar risk. Because serious complications appear rare, observing, and/or rescanning all patients with either of these medications can be debated. Risk stratification of these patients might have the potential to identify the patients at risk while safely reducing observation times and rescanning.

## Introduction

Traumatic brain injury (TBI) causes major worldwide morbidity, mortality, and accounts for approximately 3% of emergency department (ED) visits [1]. Up-front assessment of TBI severity and outcome is complex because initial signs and symptoms do not always correlate with the extent of injury [2, 3]. Presently, computerized tomography (CT) scan of the head is the gold standard in TBI to detect important injuries and the diagnostic accuracy is almost perfect [4]. In 5–10% of TBI patients, a traumatic intracranial hemorrhage (TICH) is detected at the first scan [5]. However, a small portion of TICHS is not diagnosed at index-CT and denoted as delayed intracranial hemorrhages (DICHs) [6].

The definition of DICH varies, but it is usually divided into an intracranial hemorrhage found within 24 h after trauma or between 2 and 30 days [7, 8]. The risk of DICH in patients with an intact coagulation system is considered low but higher in patients with anticoagulation therapy (ACT) such as warfarin or direct oral anticoagulants (DOACs). Several studies report the risk of DICH in patients with ACT to be 0.2–1.5% [9–13]. However, the risk of DICH in patients with antiplatelet therapy (APT) is less well studied and has been reported at similar or higher levels as in patients with ACT (1.3 vs. 1.4%) [13, 14].

Current guidelines such as the New Orleans Criteria (NOC), the National Institute for Health and Care Excellence (NICE) guidelines, the Canadian CT Head Rule (CCHR), Eastern Association for the Surgery of Trauma (EAST) practice management guidelines, and the Scandinavian Neurotrauma Committee Guidelines (SNC) take risk of TICH for patients on ACT into account by mandating a head-CT [15–19]. The EAST and SNC guidelines mandate both CT and observation for 24 h in head-injury patients treated with ACT [18, 19]. However, none of the guidelines recommends screening for DICH in patients on APT to the same extent as patients on ACT. The SNC guidelines recommend extra attention to patients on APT by mandating an up-front CT and discharge if this is normal [19].

Furthermore, the consequences of DICH are poorly described. A low DICH incidence of 0.5–0.7% without mortalities for patients on ACT or APT has been reported in low-energy traumas in the elderly [8, 20].

The rationale for performing this study was that the occurrence and consequences of DICH in head trauma patients in general and patients on APT/ACT in particular are inadequately explored in the current literature.

The aim was to study the prevalence and consequences of delayed intracranial hemorrhage after head trauma with a particular focus on patients with antiplatelet or anticoagulation therapy.

## Methods

Data was retrieved through a review of the medical records of patients with isolated head trauma who presented to the ED at Helsingborg General Hospital between January 1, 2020, and December 31, 2020. The ED serves a catchment population of 250,000 people and has about 45,000 visits each year. Neurosurgery is provided at Skåne University Hospital in Lund which is 40 km away. Because of this distance, patients with severe traumas that occur closer to Helsingborg General Hospital are brought there for primary interventions. The SNC guideline for TBI was used during the study period.

The inclusion criteria were as follows:

- Patients presenting to the ED with a chief complaint of “head trauma” registered in the ED information system
- Age ≥ 18 years

The exclusion criteria were as follows:

- Scheduled ED return visits
- Visits managed by a nurse without a physician’s involvement (e.g., patient with trivial trauma triaged to primary care or self-care)
- Trivial trauma (e.g., trauma to the head without trauma to the neurocranium or a small cut in need of stitches)
- Classified medical records

The following parameters were collected and analyzed in the present study:

1. Age (years)
2. Gender (male/female)
3. Age-adjusted Charlson Comorbidity Index
4. Head CT performed (yes/no)
5. Head CT outcome (hemorrhage/no hemorrhage)
6. Admission to general hospital ward (yes/no)
7. Admission to intensive care unit or neurointensive care unit (yes/no)
8. Neurosurgical intervention (yes/no)
9. Level of consciousness using Reaction Level Scale 85 (1–8)
10. Past illnesses (yes/no)
11. Anticoagulant treatment (no/vitamin-k antagonist/direct oral anticoagulant/low molecular weight heparin)
12. Platelet inhibitor treatment (no/acetylsalicylic acid (acetylsalicylic acid)/clopidogrel/ticagrelor/prasugrel/dipyramidol/combinations)
13. Other medication (yes/no)
14. New focal neurological deficits (yes/no)
15. Nausea (yes/no)
16. Vomiting (yes/no)
17. Amnesia, type, and duration (yes/no, antegrade/retrograde, time hh:mm)
18. Loss of consciousness (yes/no)
19. Peritraumatic seizure (yes/no)
20. Trauma mechanism

One patient could be included several times in the study if there was a new trauma. If a new trauma occurred and an intracranial hemorrhage was found on a CT after this, it was recorded as a primary traumatic intracranial hemorrhage.

The primary outcome measure was the rate of delayed intracranial hemorrhages. Secondary outcome measures were deaths and interventions such as intensive care or neurosurgical operations because of the head trauma.

Comorbidity and age were quantified with the validated age-adjusted Charlson Comorbidity Index (aa-CCI) [21–24].

All hospitals in the Skåne Region area share electronic medical records. ED records, medical records from the ward (if admitted), and radiology reports were examined from the entire geographic region. Records up to 1 year prior to the ED visit were searched for ongoing medication and comorbidities that were omitted in the ED records. No death records were reviewed which might have led to patients that died at home or in nursing care facilities being missed. Missing data from the physician’s report was retrieved, when possible, through a review of nurses’ notes and ambulance reports. Screening of medical records in Region Skåne within 6 months after index visit was performed for follow-up.

Data was collected by two reviewers. Reduction of information bias was attempted by adhering to guidelines for retrospective medical record reviews [25]. This entailed setting up and following an inclusive pro-forma document stating how data interpretation and coding should be performed and formulating well-defined criteria for inclusion and exclusion ahead of information collection. A Cohen’s kappa analysis of 100 randomized medical records reviewed by two researchers was previously published with good or very good agreement in all parameters but “new neurological deficits” and “LMWH-treatment” [26].

### Data definitions and missing data

Index-CT was defined as the CT performed at the first ED visit after the head trauma. Patients were rescanned with further CTs as per clinical indication (e.g., a patient was discharged and came back with worsened symptoms or was admitted but did not recover as expected). TICH was defined as intracranial hemorrhage diagnosed by index-CT. DICH was defined as intracranial hemorrhage diagnosed after the initial ED index visit that did not detect TICH (i.e., index-CT not detecting TICH or patient discharged from ED without CT). A distinction between delayed intracranial hemorrhages diagnosed within 24 h of trauma and 2–30 days was done as those diagnosed after 24 h would not have been diagnosed during the mandated 24-h observation. Absence of TICH was defined as “no TICH identified on any CT within 30 days or/and patients discharged from ED without CT prescribed”. The intervention was defined as neurosurgical intervention or intensive care of any sort because of head trauma. Death due to TBI was defined as death within 30 days from an index visit attributed to the head injury. APT was defined as one or more antiplatelet therapies (acetylsalicylic acid, clopidogrel, ticagrelor, prasugrel, or dipyramidol) and no simultaneous ACT or low molecular weight heparin (LMWH) therapy. ACT was defined as any oral pharmaceutical inhibiting coagulation factors (Warfarin, Apixaban, Dabigatran, Rivaroxaban, or Edoxaban) and no simultaneous APT or LMWH treatment. Patients who were prescribed both ACT and APT were analyzed separately and patients prescribed double APT were included in the APT cohort. Prothrombin international normalized ratio was not routinely investigated in the head trauma patient category and could therefore not be included in this study.

Missing data was coded as such and analyzed as the absence of findings (e.g., if vomiting was not mentioned, the parameter was analyzed as “no episodes of vomiting”). This was based on clinical judgment and experience in that ED physicians produce pragmatic medical records and only mention positive findings. The parameters we extracted are typically evaluated when assessing TBI patients and this way of dealing with missing data was considered an acceptable risk of systematic information bias. We have published previous TBI studies handling missing data this way [1, 26, 27].

Using Reaction Level Scale (RLS) is common practice in Sweden, and RLS was converted to GCS. Previous research has shown a good correlation between RLS1-2 and GCS14-15 but discrepancies between RLS3 and GCS13-8. To make study results internationally valid but still scientifically correct, the level of consciousness was only reported as GCS15-14 and GCS < 14 [28].

### Statistical analysis

Data analysis was performed with SPSS version 27 for Mac. Histograms and Shapiro-Wilks formula were used to explore data distribution. Non-parametric data was presented with median, and 25th and 75th percentiles (Q1 and Q3). The distribution of incidence of DICH was quantified with a confidence interval (CI) according to the Poisson method. Differences in clinical traits between patients with TICH and DICH were tested with Fisher’s test when appropriate or Mann–Whitney *U* test when appropriate. *P* < 0.05 was used for statistical significance.

Because of the low number of DICHs in the present material, a medical statistician was consulted. It was decided that we would only present univariate statistical analyses, even if the conclusions that could be drawn from this would be modest at best.

## Results

**Fig. 1:**
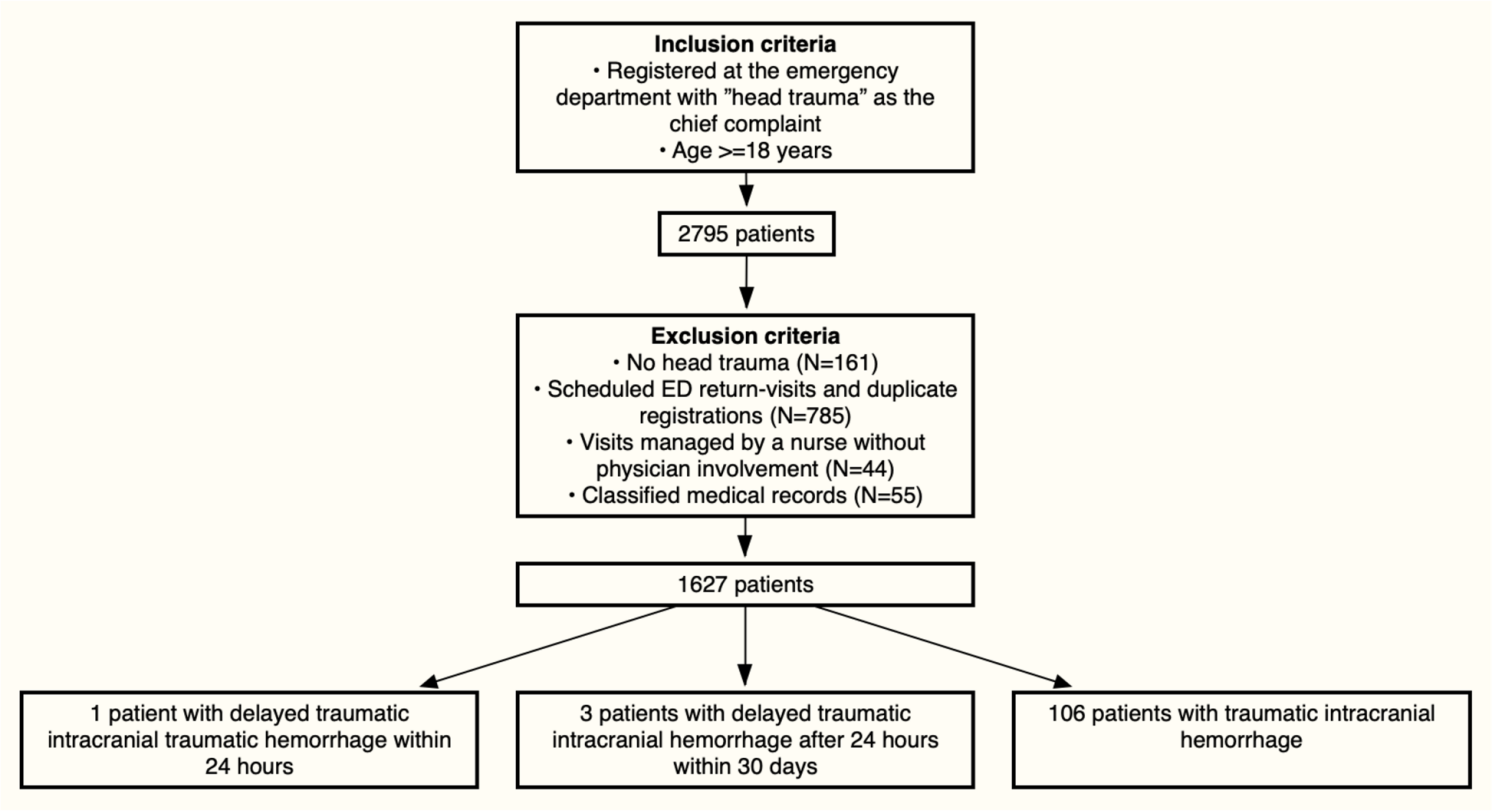
Inclusion process. This shows inclusion and exclusion criteria, the number of patients that were excluded in each step, and the number of patients with different types of traumatic intracranial hemorrhage

A total of 1627 patients with head trauma were included in the present study. See Fig. 1 for the inclusion process and distribution of intracranial hemorrhages.

An index-CT was performed on 1177 (72.3%) patients, and a total of 450 (27.7%) patients did not receive an index-CT. TICH was found in 106/1627 (6.5%) patients. Of the 1521 patients not diagnosed with TICH, 10 (0.65%) patients were rescanned within 24 h, yielding 1 DICH. Of the 1511 patients not diagnosed with TICH or DICH within 24 h, 47 (3.1%) patients were rescanned within 2–30 days, yielding another 3 DICHs. In total, DICH was found in 4/1627 (0.25%, CI 0.06–0.64%) of all included head trauma patients. Please see Table 1 for the description of the anatomic locales of the different intracranial hemorrhages.

**Table 1:** Localization of intracranial hemorrhages

Type of intracranial hemorrhage	Delayed intracranial hemorrhage within 24 h (*N*^a^ = 1)		Delayed intracranial hemorrhage within 30 days (*N* = 3)	Traumatic intracranial hemorrhage (*N* = 106)
Acute subdural hematoma	1	-		19
Subacute/chronic subdural hematoma	-	-		10
Subarachnoid hemorrhage	-	1		15
Intracerebral hemorrhage	-	1		8
Cerebral contusion	-	-		8
Mixed type hemorrhage	-	1		27
Not clearly described in the radiology report	-	-		19

^a^*N* number

The number of patients in the entire cohort with ACT was 386 (23.7%), of which 108 had vitamin-K antagonist treatment and 278 had DOAC treatment. Antiplatelet therapy was found in 256 (15.7%) patients, of which 205 had ASA treatment, 40 had other single APTs, and 11 had dual APT. Please see Table 2 for descriptive data and Table 1 for data on anatomic localization of intracranial hemorrhages.

**Table 2:** General patient characteristics

Variables	All patients
	**(*n* = 1627)**
**General patient characteristics**	
Age (median (quartile 1, quartile 3)) years	72 (49, 84)
**Gender**	
Female	822 (50.5%)
**Glasgow Coma Scale (GCS)**	
GCS 14–15	1558 (95.8%)
GCS < 14	15 (0.90%)
**Patient history**	
Vomiting	114 (7.0%)
Loss of consciousness	452 (27.8%)
Amnesia	377 (23.2%)
**Charlson Comorbidity Index**	
Median (quartile 1, quartile 3)	3 (1, 5)
0–5	1271 (78.1%)
6–14	356 (21.9%)
**Clinical findings**	
New neurological deficits at the index visit	101 (6.2%)
Peritraumatic seizures	27 (1.7%)
Traumatic intracranial hemorrhage diagnosed at index-computerized tomography	106 (6.5%)
**Clinical measures**	
Index head computerized tomography performed	1177 (72.3%)
Admission to hospital	590 (36.3%)
Intervention^a^ during index visit	7 (0.43%)
Death due to head injury	10 (0.61%)
All-cause mortality	36 (2.2%)

^a^Intensive care of any kind or neurosurgical operation

The patient was diagnosed within 24 h and one of the patients diagnosed within 2–30 days had APT. None of the patients with DICH was prescribed ACT. Please see Table 3 for a comparison of patients with TICH and DICH. The difference between ACT and APT in patients diagnosed with DICH and patients not diagnosed with DICH was not statistically significant (*p* = 1.0 and *p* = 0.578, respectively).

**Table 3:** Comparison of patients with traumatic intracranial hemorrhage diagnosed at index visit and patients with delayed intracranial hemorrhage

Variables	Patients with delayed intracranial hemorrhage	Patients with traumatic intracranial hemorrhage diagnosed at the index visit	*P* value
	**(*n* = 4)**	**(*n* = 106)**	
**General patient characteristics**			
Age (median (quartile 1, quartile 3)) years	64 (60, 71)	79 (70, 87)	0.069^a^
Female	2 (50.0%)	48 (45.3%)	1.0^b^
**Glasgow Coma Scale (GCS)**			
GCS 14–15	4 (100%)	102 (96.2%)	1.0^a^
GCS < 14	0 (0%)	4 (3.8%)	1.0^a^
**Patient history**			
Vomiting	1 (25%)	10 (9.4%)	0.348^b^
Loss of consciousness	3 (75%)	17 (16.0%)	0.462^b^
Amnesia	3 (75%)	33 (31.3%)	0.364^b^
Peritraumatic seizures	0 (0%)	1 (0.9%)	1.0^b^
**Charlson Comorbidity Index**			
Median (quartile 1, quartile 3)	3 (1, 4)	4 (3, 6)	0.143^b^
**Medications affecting coagulation**			
Oral anticoagulation therapy^c^	0 (0%)	31 (29.2%)	0.575^b^
Oral antiplatelet therapy^d^	2 (50%)	28 (26.4%)	0.299^b^
**Clinical findings**			
New neurological deficits	0 (0%)	18 (17.0%)	1.0^b^
**Clinical measures**			
Treatment at intensive care unit	0 (0%)	7 (6.6%)	1.0^b^
Neurosurgical operation	0 (0%)	3 (2.8%)	1.0^b^
Death due to intracranial hemorrhage	0 (0%)	7 (6.6%)	1.0^b^

^a^Fisher’s test. ^b^Mann-Whitney *U* test. ^c^Includes warfarin, apixaban, dabigatran, rivaroxaban, and edoxaban. ^d^Includes acetylsalicylic acid, clopidogrel, ticagrelor, and dual antiplatelet therapy

None of the DICH patients died, nor were any neurosurgical interventions performed, and all but one of these patients were admitted at index visits. All patients with DICH had index CTs performed that did not show any TICH. The patient that developed DICH and was initially discharged from the ED was not on either ACT or APT.

## Discussion

The present study was a retrospective medical records review of adult patients seeking the ED with a head injury as the chief complaint. It included 1627 patients of which 0.25% (95% CI 0.06–0.64%) were diagnosed with DICH. This rate is at the low end of what has been reported in other studies (0.2–1.5%) [9–13, 20]. The small number of DICHs makes it difficult to draw anything but very cautious conclusions but some observations can be made.

The most important finding was the low prevalence of DICHs in combination with the absence of intensive care, neurosurgical interventions, or deaths in patients with DICH. Even though the cohort size of the present study was rather large, providing sound statistical evidence for such rare events will require a larger sample size. Because of the design of the present study, it is impossible to know how many DICHs were missed, possibly explaining why the DICH rate is lower than some of the previous studies. However, because the medical records were screened for hospitalizations 6 months after the initial trauma, we believe that we would have found most DICHs with hospitalization as a consequence. Thus, if serious consequences truly are rare, the in-hospital observation despite a normal index-CT recommended by current guidelines to exclude DICH in patients with ACT could be reconsidered. The best way to investigate this properly would be in a multi-center setting to achieve the sample size required. Additionally, the insipient chronic subdural hematomas that generally present a few weeks after the trauma were not found in any of the patients with DICH. An explanation for this might be that they usually present with symptoms of impaired cerebral function and that the initial head trauma is so mild that they never seek medical attention because of it. Because the present study design only finds patients with “head trauma” as the presenting complaint, these patients would be missed [29].

There was no significant difference in DICH/TICH rate between patients with ACT and APT in the present study. However, the low prevalence of DICH precludes us from drawing any real conclusions from this finding. Current TBI guidelines recommend more rigorous work-up in patients with ACT, a statement that we can neither corroborate nor invalidate based on our findings. Nevertheless, only a few studies on this subject exist and they have shown a similar risk for patients on ACT and APT to develop DICH [13, 30]. To enhance the current literature, even studies such as this with few DICHs are important to publish. The DICH rate of the present study did not allow for meaningful analysis of the risk conferred by subtypes of ACT or APT or any combination of those. However, Colombo et al. (2021) showed that the risk of DICH was elevated in patients with double APT but not monotherapy [31]. Results of this and other studies imply that patients on ACT and APT should probably be considered to have a similar risk of developing DICH [13, 30]. This is not the case in any of the current international guidelines [15–19]. It is feasible that managing these patient cohorts in the same way would increase ICH detection. However, it is also conceivable that many patients would need investigation in order to find very few DICHs. If the consequences of them are as mild as this and other studies imply, such management is debatable [12, 13, 32]. Risk stratification based on, e.g., patient history, trauma mechanism, signs, and symptoms would be beneficial to identify the patients at risk. Thus far, only one study offers a risk stratification tool for DICH and it has not been externally validated [9]. It found that old age, craniofacial injury, neck injury, diabetes, and hypertension were associated with DICH. Further studies on risk factors for DICH would probably have the potential to greatly reduce CT-rate in TBI patients [20].

Even if almost a fourth of the patients in the present study had ACT, the absence of DICH in this cohort is not enough evidence to discourage the mandatory Scandinavian 24-h observation after head trauma. However, this finding mandates deliberation and this long observation period has been disputed in the past [33–35]. Vershoof et al. (2018) argued that all significant DICHs in patients with ACT could be found on index-CT when properly scrutinized and that only 0.2% of the patients developed DICHs that were found within 24 h [10]. It has been argued that the constant improvements of CTs will mean that more and more intracranial hemorrhages are found at index-CT and that the rate of DICHs will continue to decrease [36]. Additionally, the rescanning of patients with small TICHs is an entirely different issue and should not be confused with the primary investigation of patients in order to rule out DICH.

The low yield of mandatory CT scans and observation to find DICH carries substantial costs. It has been estimated to cost $1,000,000 to find one DICH [37]. Furthermore, not all DICHs need intervention which entails that the cost to find a DICH in need of intervention is even higher. It can be difficult to use fiscal arguments against diagnosing DICHs but this still needs to be taken into account, at least by the governing bodies of health care. Resources used to observe and rescan to rule out DICH can potentially delay care for other patients in the ED that have more urgent medical needs. Moreover, many of the patients on APT or ACT are older and frail. It has been shown that even shorter terms of hospitalization might have a negative impact on their life span [38]. Thus, avoiding unnecessary admissions in these individuals is important not only from an economic point of view.

The retrospective method is the most salient limitation of the current study. Information bias can occur when scrutinizing medical records and dealing with missing data. Notwithstanding efforts to counter this, only careful conclusions can be drawn from this study. Our pragmatic way of dealing with missing data was debated in the study group prior to data collection and considered the best solution. However, it carries the disadvantage of precluding reliability measurements such as confidence intervals. The direction of bias can therefore not be quantified.

Another source of bias is the patients’ compliance with taking their prescribed medication affecting coagulation. It is possible that this affects the results of this study but the magnitude of that bias cannot be quantified with the current study design.

Construing “Head-CT not performed” as the absence of intracranial hemorrhage can lead to missed intracranial hemorrhages. Nonetheless, because we searched the medical records for ED visits 6 months after the index visit, it can be assumed that intracranial hemorrhages with severe consequences would have been found. However, the true rate of DICHs cannot be found with this methodology. If patients die in their homes because of DICH, they are also missed.

## Conclusion

DICH after head trauma seems rare and none of these patients died or needed intensive care or neurosurgery. APT and ACT might confer similar risks of DICH. Because serious complications to DICH appear rare, observing and/or rescanning all patients on ACT or APT can be debated. Risk stratification of these patients might be a way to identify the patients at higher risk while safely reducing observation times and rescans.

## Data Availability

Data will be made available upon request.
